# Silicosis With Secondary Spontaneous Pneumothorax in the Western Rajasthan

**DOI:** 10.7759/cureus.11811

**Published:** 2020-11-30

**Authors:** Manish Kumar Meena, Romil Singh, Nalin Joshi, Sawai Singh Rathore, Sindhu Chadalawada, Malik Abubakar, Shruthi Badam, Kaushal Shah

**Affiliations:** 1 Respiratory Medicine, Sawai Man Singh Medical College, Jaipur, IND; 2 Internal Medicine, Metropolitan Hospital, Jaipur, IND; 3 Internal Medicine, Dr. Sampurnanand Medical College, Jodhpur, IND; 4 Internal Medicine, Mahatma Gandhi Medical College and Research Institute, Pondicherry, IND; 5 Medicine, Our Lady of Fatima University, College of Medicine, Valenzuela, PHL; 6 Internal Medicine, Osmania Medical College, Hyderabad, IND; 7 Psychiatry, Griffin Memorial Hospital, Norman, USA

**Keywords:** silicosis, primary pneumothorax, secondary pneumothorax, pneumoconiosis

## Abstract

Objective

Silicosis is one of the common occupational lung diseases caused by crystalline silica respiration. Pneumothorax is one of the most common and morbid complications of silicosis involving lung pleura. It is commonly seen unilaterally in chronic silicosis and can often be lethal. The purpose of this study is to report secondary spontaneous pneumothorax (SSP) in critically ill patients with silicosis.

Methods

A cross-sectional study was done between January 2019 and June 2019 at Sawai Man Singh (SMS) Medical College in Jaipur, India. A cohort of 50 patients with dyspnea and a history of silicosis were studied. A chest X-ray and sputum for acid fast bacilli were checked on all suspected cases.

Results

The present study showed that the mean age of patients was 38.7 years, all silicosis patients had dyspnea, and 96% of patients had severe chest pain. The results of chest X-rays concluded the evidence of silicosis. Bilateral pneumothorax was seen in three cases, right-sided pneumothorax in eight cases, and left-sided pneumothorax in 11 cases. The rate of pneumothorax incidence in silicosis patients was about 44%, which is higher than the current evidence. Six patients were managed conservatively with oxygen and bronchodilators, and 16 patients underwent through tube thoracostomy.

Conclusion

This study highlights the importance of considering spontaneous pneumothorax in patients who are presenting with shortness of breath and/or chest pain especially with a known history of silicosis, as the timely diagnosis can alter the management of this morbid condition which carries a high mortality rate if left untreated, compromising the lung expansion, venous return, cardiac output, oxygenation and eventually leading to death.

## Introduction

Silicosis is an occupational lung illness due to the inhalation of crystalline silica. The International Agency for Research on Cancer (IARC) has listed crystalline silica as a “group 1” carcinogenic agent [1]. This typically occurs in the lungs after decades of silica exposure, and it transforms progressively into nodular fibrosing pneumoconiosis [2]. Silicosis has impacted many countries across the globe, including China, Brazil, the United Kingdom, the USA, Iran, and India [3,4]. In certain occupations, workers are exposed to high silica concentrations, causing dysfunction of lungs due to the fibrogenic process. Since silica is odorless and does not cause immediate lung irritation, a large exposure of it can possibly not be noticed or observed. Crystalline versions of silica are more fibrogenic than amorphous ones, stressing their significance and their role in silicosis pathogenesis. The disease has a long dormant period and hence can appear clinically in the late phase. The occurrence of silicosis disease is certainly among those with silica exposure, posing a critical health problem in these populations, especially in low-income communities [4].

In silicosis, pleural consequence, like pleural effusion, pleural rigidity, or pneumothorax, is hardly seen. Pneumothorax is indeed one of the significant complications of pleura-related silicosis. It is typically observed unilaterally in chronic silicosis and can be lethal at times. However, pneumothorax is uncommon as an acute manifestation of silicosis. Usually, silicosis does not impact the lung pleural adversely, except increasing the risk of silicosis is secondary spontaneous pneumothorax (SSP). The SSP is detected late in the disease and can be lethal in combination with the underlying grossly impaired lung function. SSP is typically unilateral but may sometimes occur as a bilateral pneumothorax condition [2,3]. As the incidence and prevalence of SSP in silicosis is understudied, especially the bilateral pneumothorax, it needs further research. The purpose of this research is to understand and study SSP in silicosis patients.

## Materials and methods

We reviewed the clinical and imaging records of 50 silicosis patients complaining of dyspnea at the Sawai Man Singh (SMS) Medical College of Jaipur in India. Exclusion criteria included age less than 18 years, history of trauma, and HIV positive patient. All participants provided consent and enrolled in this cross-sectional study. Occupational history was collected in detail from all patients, including types of stone mine, location, activity information, duration of exposure of dust or silica particles before being included in the study. Participants also provided medical history and imaging studies, such as chest radiography. Sputum was tested on all cases for acid-fast bacilli through Ziehl-Neelsen (ZN) staining. In all these cases, pneumothorax was clinically diagnosed by auscultation and later confirmed radiologically by a recent chest X-ray.

## Results

The present study revealed that the mean age of patients was 38.7 years, and most patients were in 20 to 30 years of age group (Table 1).

**Table 1:** Distribution of patients by age SD: Standard deviation; %: Percentage

Age groups	Number of patients	Percentage
20-30 years	18	36%
31-40 years	12	24%
41-50 years	14	28%
>50 years	6	12%
Total	50	100%
Mean ± SD	38.70 ± 10.17 years	

All silicosis patients complained of dyspnea, and 96% of patients had severe chest pain graded on the basis clinical history given by patients (Table 2).

**Table 2:** Chief complaints of patients %: percentage

Chief complaint	Number of patients	Percentage
Dyspnea	50	100%
Chest pain	48	96%
Dry cough	29	58%
Productive cough	17	34%
Cough with expectoration	4	8%
Fever	25	50%
Loss of appetite	20	40%
Loss of weight	15	30%

The mean duration of exposure to silica particles was 13.82 years, with a range of five years to 24 years (Table 3).

**Table 3:** Exposure to silica particles SD: Standard deviation

Duration of exposure	Statistics
Mean	13.82 years
SD	4.762 years
Minimum	5 years
Maximum	24 years
Range	5-24 years

The radiographic imaging of lungs through chest X-rays implied silicosis. Bilateral pneumothorax was seen in three cases, unilateral right-sided pneumothorax in eight cases, and unilateral left-sided pneumothorax in 11 cases. The pneumothorax incidence rate in the patients of silicosis is about 44%, higher compared to the currently available evidence (Table 4).

**Table 4:** Chest X-ray findings N: number of cases; %: percentage

Chest X-ray findings		Number of patients	Percentage
Suggestive of silicosis		28	56%
Pneumothorax (N = 22)	Bilateral	3	13.63%
	Left	11	50%
	Right	8	36.37%

**Figure 1:**
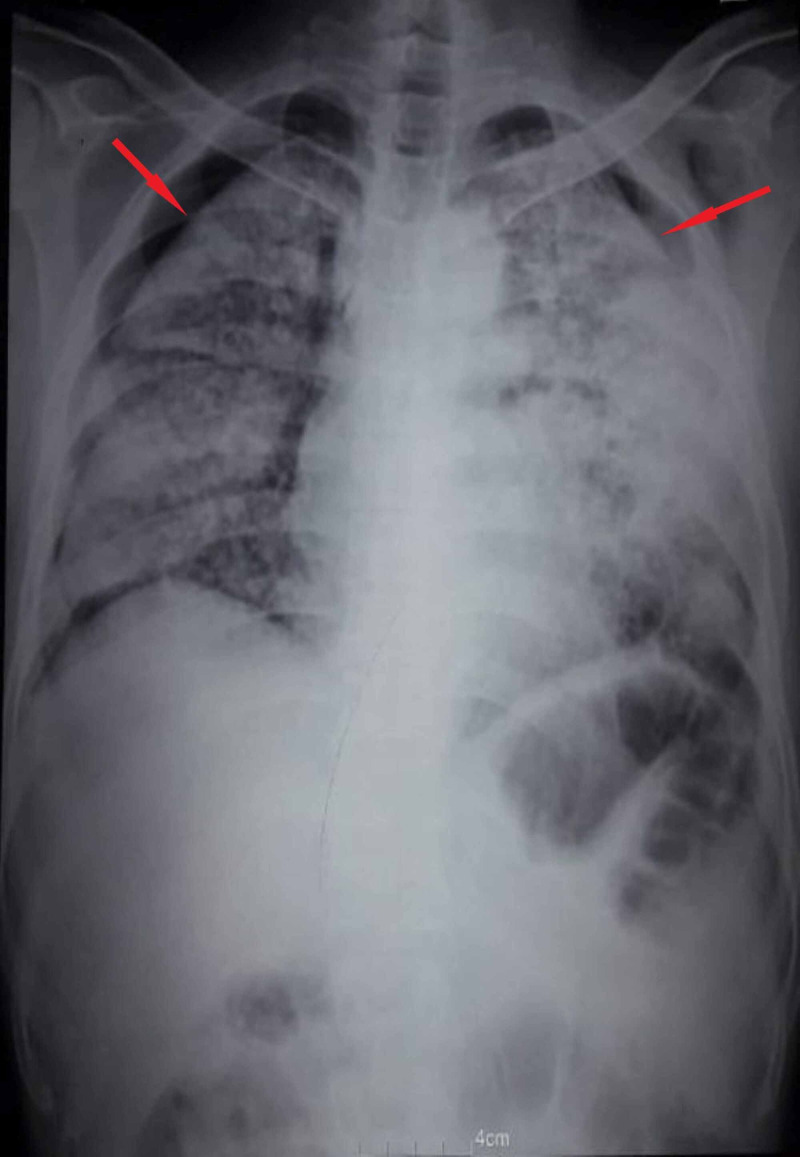
Chest X-ray of bilateral pneumothorax

Bilateral pneumothorax, as seen in Figure 1.

**Figure 2:**
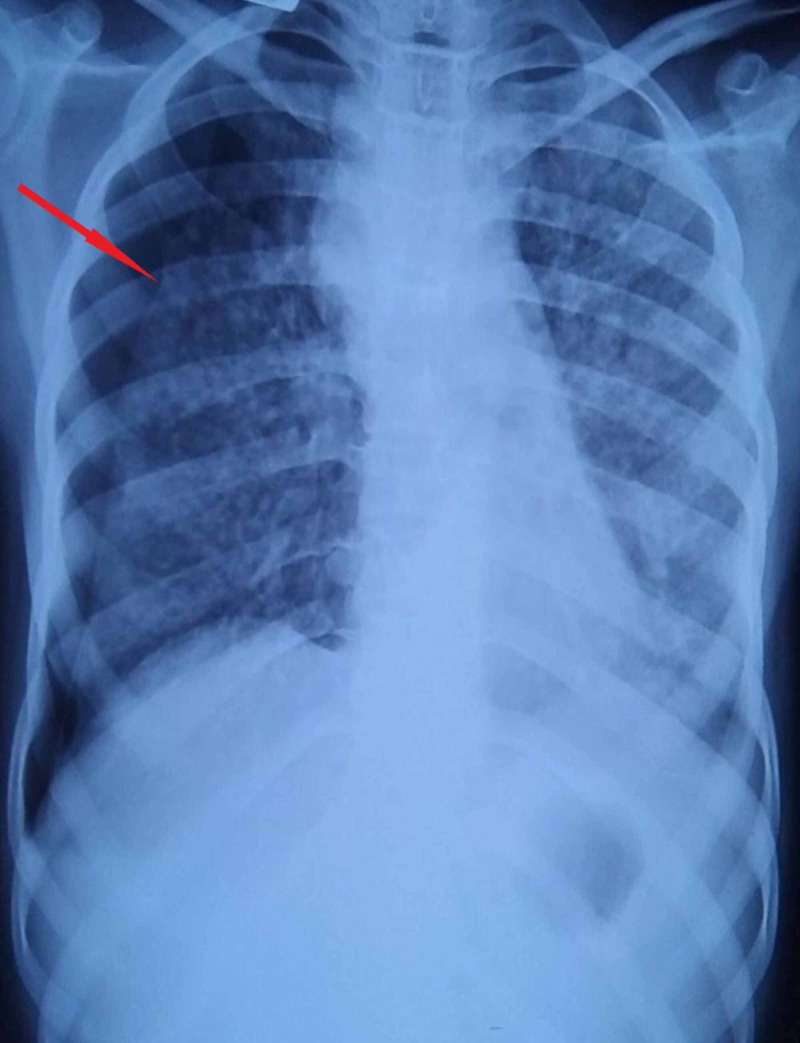
Chest X-ray of unilateral right-sided pneumothorax

Unilateral right-sided pneumothorax, as seen in Figure 2.

**Figure 3:**
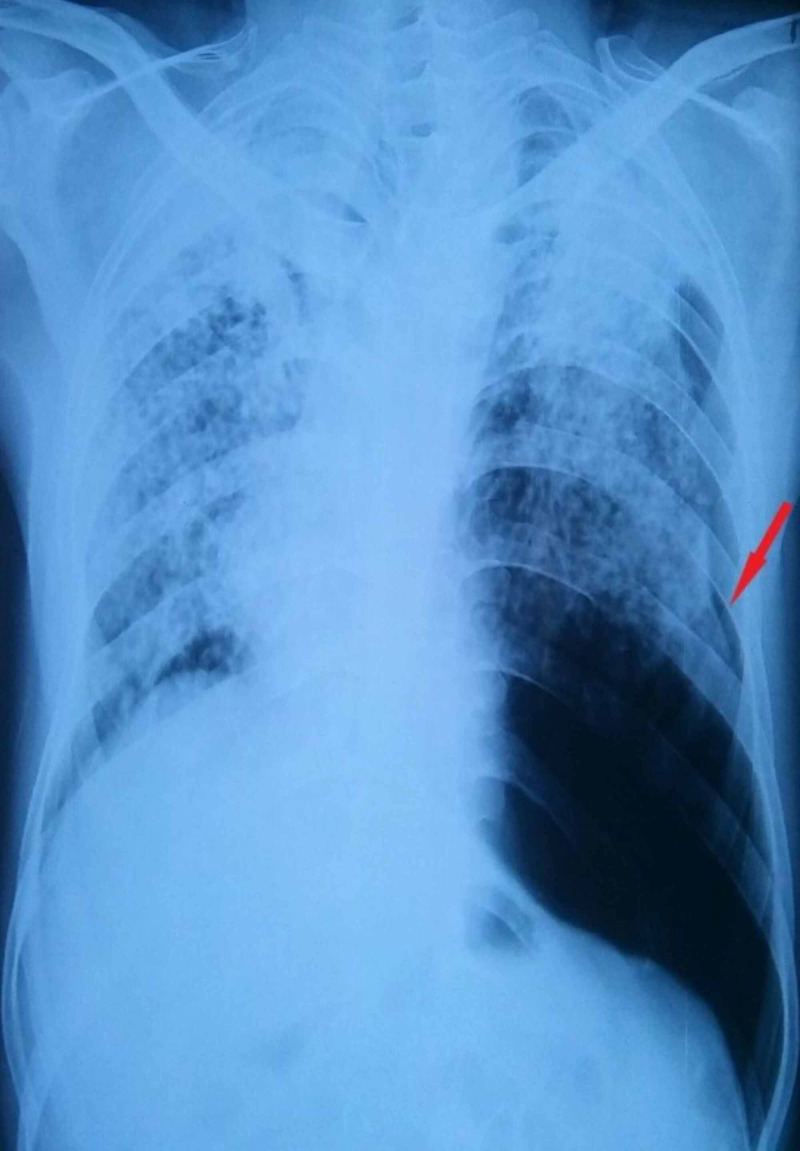
Chest X-ray of unilateral left-sided pneumothorax

Unilateral left-sided pneumothorax, as seen in Figure 3.

The pneumothorax patients were all identified smokers and on average they smoked 16 packs a year (Table 5).

**Table 5:** History of smoking %: percentage

History of smoking	Number of patients	Percentage
Non-smokers	12	24%
Smokers	38	76%
Total	50	100%

Most cases working in manual stone crushing (40%) and reside in Jodhpur (42%) (Tables 6, 7).

**Table 6:** Occupation wise distribution of patients %: percentage

Occupation	Number of patients	Percentage
Crusher machines	19	38%
Mining	11	22%
Stone crushing	20	40%
Total	50	100%

**Table 7:** Types of stones %: percentage

Type of stones	Number of patients	Percentage
Jodhpur	21	42%
Karauli	15	30%
Dholpur	14	28%
Total	50	100%

The intervention of tube thoracostomy was done in 16 patients. About six cases were managed conservatively with oxygen and bronchodilators who had a minimal pneumothorax (Table 8). The average hospital length of stay was 11 days.

**Table 8:** Treatment of complication of pneumothorax in silicosis patients ICDT: Intercostal drainage tube; %: percentage

Treatment of pneumothorax	Number of patients	Percentage
Bilateral ICDT	2	9.09%
Left ICDT	8	36.36%
Right ICDT	6	27.27%
Conservative	6	27.27%
Total	22	100%

Silica was found highest in Dholpur stone, followed by Jodhpur and Karoli stone (Table 9).

**Table 9:** Chemical composition of different sand stone SiO_2_: Silicon dioxide; Fe_2_O_3_: Ferric oxide; Al_2_O_3_: Aluminium oxide; CaO: Calcium oxide; MgO: Magnesium oxide; LOI: Loss of ignition.

Chemical composition in percentage	Jodhpur	Karoli	Dholpur
				SiO_2_	96.6	96.2	98.2
Fe_2_O_3_	1.2	0.8	0.84
Al_2_O_3_	1	1.2	0.32
CaO	0.28	0.4	0.28
MgO	0.2	0.2	nil
LOI	0.5	0.6	0.2

Density was found highest in Jodhpur stone, followed by Dholpur and Karauli stone (Table 10).

**Table 10:** Technical information on sandstone kg/m^3^: kilogram per cubic meter; %: percentage; kg/cm^3^: kilogram per cubic centimeter

Properties	Jodhpur	Karauli	Dholpur
Density (kg/m^3^)	2.42	2.38	2.4
Water Absorption (%)	1.25	1.2	1.2
Modulus of Rupture (kg/cm^3^)	220	210	208
Compressive Strength (kg/cm^3^)	390	358	460

## Discussion

Silicosis usually occurs in workers involved in the design, mining, sand, tunneling, discovery work, and ceramics industry [5]. Acute crystalline silica is about 10 microns thick and can easily reach the lower parts of the lung. It accumulates in the lower respiratory system even though the exposure of silica is little [6]. Several studies have shown the association of secondary silicosis pneumothorax with the presence of bullae [3]. Due to direct silica's toxic damage on the lungs, inflammatory products formed damage the alveolar wall's elastic fibers that contribute to bleb creation [7]. Severe lung fibrosis develops due to silicosis. It also causes secondary autoimmune pneumothorax through the rupture of bullae because of the inability to detect weak lung parenchyma in the early stages [3]. Other reproductive disorders and the emergence of type II cells are also thought to lead to pneumothorax proliferation [7].

A co-existing disease of tuberculosis also expedites the process of developing fibrosis in silicosis patients. In endemic countries, this disorder could be found in 20 to 25% of silicosis patients across their life. Other complications include esophageal stress and cor pulmonale. Crystalline silica is considered to be more pathogenic if it is less than 1 cm in size [8]. Primary tissue damage by silica particles induces inequities between inflammatory reaction generation. It leads to decreased alveolar walls elasticity. The development of pneumothorax can be characterized by a lack of activation of type 2 alveolar cells. Pneumothorax happens typically due to chronic exposure of silicosis and could progress into fibrosis. In aggressive silicosis, sporadic spontaneous pneumothorax cases have been documented [8].

The factors contributing to the development of secondary spontaneous pneumothorax (SSP) in silicosis patients have not been studied yet in detail. A study showed a close correlation between SSP incidence and bullae appearance. Silicosis is also associated with morphological changes in the lungs, besides smoking. In advanced silicosis, a coalescence of perinodular emphysematous areas can lead to macroscopic blebs' formation, and its eruption causes pneumothorax [9].

The silica's chemical and physical properties could have an association with pneumothorax, as silicon dioxide is high amongst patients of Dholpur, Karoli, and Jodhpur. The stone in this area is a sandstone that is defined as a stone made of quartz grains and other minerals of the same size and is usually smooth and round. Quartz, cristobalite, and some tridymite modes are piezoelectric. Piezoelectricity is a phenomenon in which direct force exerted to the crystal generates different electrical charges on the contrary sides of the body structure. This is the case in crystalline silica as the molecular structure has no center, exhibiting internal differences. In addition, the opposite sides of these particles have different faces and carry different electrical charges. It is known that the pathophysiology of silica-related disease may be influenced by the production of free oxygen radicals on the silica molecular surface and by the impact of weakened alveolar macrophages [10,11].

In our research, we studied 21 cases of Jodhpur stone, 15 cases of Karauli stone, and 14 cases of Dholpur stone. Also, the Jodhpur stone is denser than Karauli and Dholpur stone. It indicates the role of stone density in causing silicosis. Our study found a higher number of secondary pneumothorax cases amongst smokers with silicosis. It confirms and corroborates the research findings conducted by Bense et al. [12]. This study also reflects the previous findings of a higher prevalence of silicosis in the younger population [13].

As in other instances of spontaneous secondary pneumothorax, silicosis-induced pneumothorax requires an assertive treatment strategy. There is, still, no negotiated agreement on the therapy. The tube thoracostomy intervention is recommended for all cases, especially in the first instance, when the risk of recurrence is higher due to severe inflammation and fibrosis.

## Conclusions

This study highlights the importance of considering spontaneous pneumothorax in patients who are presenting with shortness of breath and/or chest pain especially with a known history of silicosis, as the timely diagnosis can alter the management of this morbid condition which carries a high mortality rate if left untreated, compromising the lung expansion, venous return, cardiac output, oxygenation and eventually leading to death. Our analysis of silicosis patients found an increased incidence of secondary pneumothorax. Smoking may be a major contributor to pneumothorax production. When pneumothorax occurs in silicosis patients, tube thoracostomy should be performed in all cases, particularly in the first episode, as there is a high recurrence chance.
